# Microbial Reference Frames Reveal Distinct Shifts in the Skin Microbiota after Cleansing

**DOI:** 10.3390/microorganisms8111634

**Published:** 2020-10-23

**Authors:** Riccardo Sfriso, Joshua Claypool

**Affiliations:** 1 DSM Nutritional Products, Personal Care, Wurmisweg 576, CH-4303 Kaiseraugst, Switzerland; 2 DSM Nutritional Products, Nutrition Innovation Center, Lexington, MA 02421, USA; joshua.claypool@dsm.com

**Keywords:** skin microbiome, microbiota, cleansing, cosmetic, skin care, reference frames

## Abstract

Skin cleansing represents a process of mechanical and chemical removal of dirt, pollutants as well as microbiota from the skin. While skin cleansing can help maintain good health, protect us from infections, illnesses and ailments, skin cleansing can also strip away lipids and moisture from the skin, leading to irritation, barrier impairment and disturbance of the delicate cutaneous microbiome. This study investigated how skin cleansing impacts skin’s microbial composition. Thirty Caucasian women were enrolled in a placebo controlled clinical study where participants applied on their volar forearms a liquid body wash twice daily for 1 week in order to mimic frequent showering. Skin microbiome samples were collected by swabbing at defined timepoints and 16S rRNA sequencing was performed. Using “reference frames”, we could identify shifts in the microbial composition and several microbiota were identified as being characteristically associated with the presence of saccharide isomerate, a well-known skin moisturizer. The microbial shift was quite immediate, and we could observe it already at 1 h post cleansing. Interestingly, the new microbial composition reached a certain dynamic equilibrium at day 1 which was then maintained until the end of the study. *Paracoccus marcusii*, a potentially beneficial carotenoid-producer microorganism, was enriched by the active treatment and, at the same time, the abundance of several potential pathogenic taxa, *Brevibacterium casei* and *Rothia mucilaginosa,* diminished.

## 1. Introduction

Skin cleansing is generally considered a chemical and mechanical process of removing dirt from the skin surface, including dust, sweat, sebum, old corneocytes and pollutants as well as potential infectious agents [1]. Nowadays, skin cleansing products such as bar soaps or liquid soaps have become an integral part of our daily body cleansing routine, which is not limited to cleaning the skin but extends to physical and psychological relaxation or pleasure resulting from a shower or a warm bath. An ideal skin cleanser should be mild to the skin, and at the same time must have an effective cleansing ability while ensuring a good sensory during use, including the ability to form a good foam which can be easily rinsed off. An efficient cleansing is guaranteed by surfactants, cleansing agents that are composed of a hydrophilic and a hydrophobic region. In an aqueous solution, surfactants organize themselves into micelles, having an internal lipophilic pocket surrounded by external hydrophilic groups. These micelles scavenge oily substances, such as sebum, soil and other contaminants from the skin and disperse them in water for removal by rinsing [2]. It is well documented in the scientific literature that cleansing agents, even simple tap water, have an impact on the biophysical properties of the skin influencing its pH, lipid content and hydration [3,4,5,6]. Surfactants are known to interact with stratum corneum proteins such as keratin and cause their denaturation, resulting in swelling of the stratum corneum [7].

The physiological skin pH—to which we normally refer to as “acid mantle” [8,9]—is known to be below the value of 5 and results from different passive and active mechanisms occurring in the skin. Among the components known to be responsible for the low skin surface pH are lactic acid, which naturally occurs in eccrine sweat and as a product of epidermal metabolic processes [10], as well as free fatty acids coming from lipase-mediated hydrolysis of phospholipids during the cornification process [11,12,13,14]. Commensal skin-resident microbiota also contributes to the maintenance of the low skin surface pH—representing the optimum for many epidermal enzymes—by secreting acidic metabolites and ensuring at the same time a defense mechanism against pathogens [7,12].

The skin microbiota is widely recognized to be of paramount importance for the maintenance of skin health. It is a dynamic ecosystem continuously exposed to intrinsic, host-related and extrinsic environmental factors which taken together contribute to its ecology [15]. Family and household contacts have been reported to have an important influence. Family members residing in the same home were shown to have closer similarities in skin commensal bacteria than individuals from different households [16]. Recent studies also show that each individual has a unique personal microbial “cloud”, emitting between 10^6^ to 10^7^ skin-associated microbes per h, resulting in a significant deposition of biomass in the built environment [17,18]. Other factors such as the ambient temperature and humidity, ventilation, co-occupancy, contact with the external environment, air quality and UV light are also contributing to the individual variation in skin microbiota colonization [15,19]. Even the amount of green areas and biodiversity in the vegetation surrounding the primary residence is an important factor shaping the composition of commensal microbiota and influencing immunotolerance [20,21]. Besides that, routine activities like showering or bathing using soaps and detergents are likely to impact the microbial composition of the skin’s ecosystem. During these routine activities like showering, body washes can come with a variety of ingredients that may or may not interact with the host’s skin microbiome. Of interest are a class of hygroscopic compounds, such as saccharide isomerate, which are known to hold onto water but can also be metabolized by the local microbiota. As the body wash, and specifically each of the minor ingredients, are in contact with the skin for a short duration, it is not known the impact that body wash and its composition can have on the microbiota. This study investigated how skin cleansing using a soap-based formula supplemented with saccharide isomerate impacts skin’s microbial composition. While traditional methods of analyzing changes in the microbiota are valid, they can be prone to high false discovery rates [22]. Following the methodology proposed by Morton et al. [23], we used reference frames to identify specific differentially abundant microbial taxa positively associated with the application of an active body wash formulation containing saccharide isomerate, a well-known cosmetic ingredient with a unique mechanism which binds to the skin used for short- and long-lasting moisturization, at all the timepoints evaluated.

## 2. Materials and Methods

### 2.1. Test Formulations

We used a base body wash formulation (placebo) and an active body wash formulation (treatment) consisting of the base formulation plus 0.5% saccharide isomerate (commercial product PENTAVITIN^®^, DSM Nutritional Products, Switzerland). Base and active formulations are outlined in Table 1.

### 2.2. Clinical Study Design

A placebo-controlled, single-blind, and randomized clinical study was conducted at Laboratoire Dermscan in Poland. Study participants gave their informed consent to participate in the study and the general principles of the Declaration of Helsinki guidelines were applied. The study was approved by the local Bioethics Committee of the Regional Chamber of Physicians in Gdansk under the project identification number #19E2742. Adverse effects were recorded.

Thirty healthy Caucasian female volunteers aged between 25 to 45 were enrolled in the study. Volunteers were encouraged to refrain from any topical application on their forearms, apart from the test products, throughout the whole study duration. Acclimatization at the test institute was performed for about 30 min before any measurements were performed.

The study consisted of two phases: A conditioning washout phase of 7 days (−168 h to 0 h) and an application phase of 7 days (0 h to 168 h). At −168 h, the study participants went to the test institute and, after acclimatization, skin microbiome samples were collected by swabbing. The volunteers were provided with a gentle cleanser to be used for 7 days, twice daily on their forearms for the whole washout phase. At 0 h, following acclimatization, baseline microbiome sampling was performed on the volar forearm skin. The first product application was then performed. Each volunteer applied the active treatment on one forearm and the placebo on the second forearm so that inter-individual variability could be reduced.

The method of product application was thought to simulate the real-life gesture of skin cleansing when taking a shower. The test site was first moistened with water and then the product in amount of 2 µL/cm^2^ was applied with a gentle massage for 20 s followed by a more energetic massage for 10 s to allow foaming. The foam was left in place for 1 min. Standardized rinsing was performed by three presses of water atomizer. The application site was dried by gently tapping the skin with a tissue for three times. During the first 24 h the products were applied only once. From 1 h after the application, skin microbiome samples were collected in an adjacent site respect to the first sampling. The measurements and samplings were repeated at 6, 24 and at 168 h (Figure S1). Starting from the second day, the products were applied twice daily by the study participants.

### 2.3. Skin Microbiome Sampling

Skin Microbiome of the stratum corneum was collected via swabbing by trained and consistent personnel who ensured that the same number of strokes and consistent pressure onto the skin was applied throughout the entire sampling procedure. Swabbing was chosen as sampling method because of its less invasive nature compared to skin scraping/tape stripping and punch biopsy and also to reduce at the minimum the sample contamination by human DNA. Skin swabs were collected from the volar forearm using Hydraflock^®^ flocked swabs (Puritan, Guilford, ME, USA) which were soaked in sterile 0.9% NaCl solution before sampling. An area of about 7cm^2^ was sampled at each time point. The swab was firmly rubbed 10 times over the sampling site for 10 s. Subsequently, the swab was placed in a 2 mL collection tube pre-filled with 1ml of DNA stabilization buffer (Zymo Research Corp., Irvine, CA, USA). The collected swab samples were stored at −80 °C until further processing.

### 2.4. qPCR

DNA was extracted from the swab samples using Zymobiomics Miniprep kit (Zymo Research Corp., Irvine, CA, USA) according to manufacturer’s instructions and incorporating bead beating for mechanical lysis. All qPCR reactions were performed in 384 well PCR plates (Thermo Fisher Scientific, Inc., Waltham, MA, USA) sealed with MicroAmp Optical Adhesive Film (Thermo Fisher Scientific, Inc., Waltham, MA, USA) using an Applied Biosystems QuantStudioTM 5 Real-Time PCR system (Thermo Fisher Scientific, Inc., Waltham, MA, USA) with QuantStudioTM Design & Analysis software v1.4.2. Each reaction for the *Staphylococcus epidermidis* or *Cutibacterium acnes* qPCR was carried out in a total volume of 10 µL, with 5 µL ABsoluteTM Blue qPCR Mix, Low ROX (Thermo ScientificTM), 0.2 µL forward primer (*S.epidermidis*: 5′-CAACTCGATGCAAATCAGCAA-3′; 10 µM; *C.acnes*: 5′-GCGTGAGTGACGGTAATGGGTA-3′; 10 µM), 0.2 µL reverse primer (*S.epidermidis*: 5′-GAACCGCATAGCTCCCTGC-3′; 10 µM; *C.acnes*: 5′-TTCCGACGCGATCAACCA-3′; 10 µM), 0.1 µL probe (FAM- TCATTTCACGCAAACTGTTGGCCACTATG-BHQ1; 10 µM), 2 µL PCR grade water and 2.5 µL undiluted template DNA. A standard curve comprising 8 serial 10-fold dilutions of a synthesized, cloned, linearized, and purified DNA of 186 bp of the femA gene (X17688.1) was generated from a work solution (0.1 ng/µL) that in turn was derived by 100 times diluting a stock solution (10 ng/µL). The PCR program started with a denaturation step at 95 °C for 15 min, followed by 40 cycles consisting of denaturation at 95 °C for 15 s, annealing and elongation at 60 °C for 1 min (with data collection). A positive control was performed alongside each separate amplification consisting of 2.5 µL of 0.8 ng/µL (2 ng gDNA added to a single reaction) that was derived from 25 ng/µL *Staphylococcus epidermidis* (DSM 20044) or *Cutibacterium acnes* (DSM 1897) gDNA. Negative template control (NTC) PCRs were performed alongside each separate amplification without addition of template. Amplification data were exported from QuantStudioTM Design & Analysis software v1.4.2 followed by determining the target quantity per µL DNA preparations using the standard curves and calculation of the number of target per unit (gram, ml, or item) of raw material using the formula below.

$$\mathrm{Quantity}\;\mathrm{per}\;\mathrm{unit}\;\mathrm{material}=\frac{\mathrm{Quantity}*D_{DNA}*V_{DNA}*\;\frac{V_{lysis}}{V_{lysis.extraction}}}{M_{material}\;or\;V_{material}\;or\;N_{material}\;}$$

Quantity: Obtained quantified target per µL DNA
$D_{DNA}$: DNA dilution factor = 1
$V_{DNA}$: Volume nuclease free water used for DNA elution = 50 µL
$V_{lysis}$: Volume reagent used for lysis in DNA extraction = 1000 µL
$V_{lysis.extraction}$: Volume supernatant after lysis used for DNA extraction = 400 µL
$M_{material},\;V_{material}\;or\;N_{material}$: Mass, Volume, or number of items material used for extraction = 1 swab

### 2.5. 16S rRNA Gene Sequencing

DNA extracted for qPCR was used for sequencing described here. The V3-V4 hypervariable region of the 16S rRNA gene was amplified using the 341F (5′-CCTACGGGNGGCWGCAG-3′) and the 785R (5′-GACTACHVGGGTATCTAATCC-3′) primers appended with Illumina adaptor sequences. The amplicons were sequenced on Illumina’s MiSeq platform with paired end 300 bp reads. Initial quality assessment was based on data passing the Illumina Chastity filtering. Subsequently, reads containing PhiX control signal were removed using an in-house filtering protocol. In addition, reads containing (partial) adapters were clipped (up to a minimum read length of 50 bp). The second quality assessment was based on the remaining reads using the FASTQC quality control tool version 0.11.5. Paired-end sequence reads were collapsed into so-called pseudoreads using sequence overlap with USEARCH version 9.2 [24]. These pseudoreads were collapsed into 97% OTUs. Classification of these pseudoreads was performed based on the results of alignment with SNAP version 1.0.23 [25] against the RDP database [26]. Taxonomic calls were based on a rank-specific identity threshold of Species 99%, Genus 97%, Family 95%, Order 90%, Class 85%, and Phylum at 80%.

### 2.6. Data Filtering and Analysis

For presentation of taxonomy and alpha/beta diversity metrics, singletons were removed from OTU-level data and then collapsed based upon taxonomic classification. Alpha and beta diversity were evaluated using Shannon’s metric and Bray–Curtis dissimilarity (scikit-bio v0.5.6).

In the use of Songbird, co-occurrence filtering methods were applied to establish shifts in the most common microbiota. A threshold of 75% was used to establish core microbiota across all participants to increase sensitivity [27]. When analyzing Songbird differentials, the top 10% of features associated with the active treatment or time differential were selected and then evaluated. The bottom 10% of features have been used as reference frames to infer changes in the microbial composition. In general, except for during the washout phase, the Songbird models were constructed only using time (numeric) and treatment (categorical).

## 3. Results

Of the total reads, an average of 96% were assembled into pseudoreads with an average read count of 48,655 per sample. Of the pseudoreads, an average of 96% were able to be classified against the RDP database.

### 3.1. Relative Abundance and Diversity

**Figure 1:**
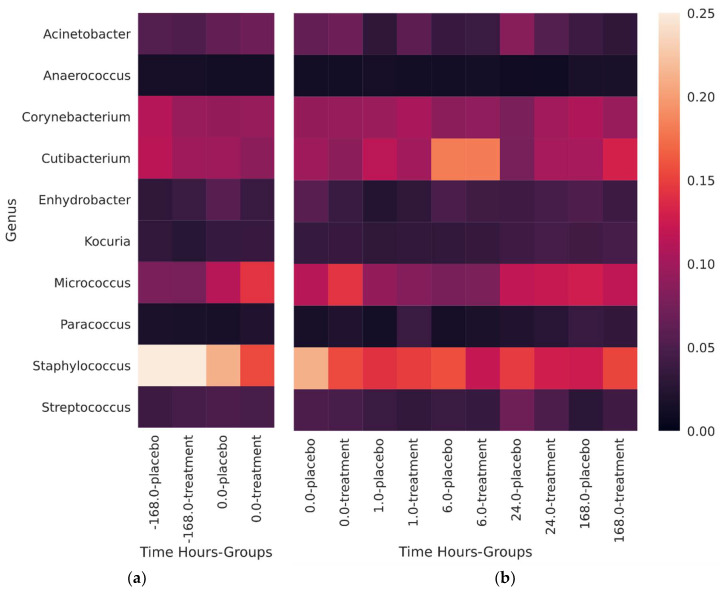
The 10 largest contributors to the relative abundance for (**a**) washout phase and (**b**) during the placebo/treatment phase. The 0 h timepoint is displayed in both (**a**) and (**b**) because it serves as a comparison for change in each of the trial segments.

The most commonly observed skin genera were *Staphylococcus*, *Micrococcus*, *Cutibacterium*, and *Corynebacterium* (Figure 1)*. Staphylococcus* was most abundant prior to the subjects starting the trial whereas *Micrococcus* were most abundant at day 0, post-washout before the application of the test products. The most abundant species of *Staphylococcus* present 7 days prior to the beginning of the study (pre-washout phase) was *Staphylococcus hominis*. The most common species of *Micrococcus* represented in the samples was *Micrococcus yunnanensis* and in much smaller proportions, *Micrococcus endophyticus*. *Cutibacterium* was present in the largest relative abundance after 6 h from the first use of the test body wash formulations. As expected, *Cutibacterium acnes* was the main contributing species to the relative abundance of *Cutibacterium* but *Cutibacterium granulosum*, *Propionibacterium namenetense*, and *Cutibacterium avidum* were also detected (listed in decreasing relative abundance). No major changes in relative abundance of *Corynebacterium* were observed across pre/post-washout phases. A wide variety of species comprised the makeup of the *Corynebacterium* genus: *C. tuberculostearicum*, *C. afermentans*, *C. jeikeium*, and *C. amycolatum* to list a few (107 different species detected in total).

*Staphylococcus epidermidis* was present at its highest average relative abundance prior to subjects joining the trial. During the first 6 h, *S. epidermidis* appeared to increase in proportion relative to time zero before stabilizing after 24 h around 4.7–5.7% of the total population. *Cutibacterium acnes* appears to remain constant in the subjects, including prior to joining the trial, at around 9.7–12.9%. Immediately after using the body wash, at one and 6 h a noticeable increase in the relative abundance was observed up to an average maximum of 17.75% relative abundance.

While no significant changes in alpha diversity were observed (Figure S2), it was generally seen that alpha diversity increased in the post-washout phase. Of note was that diversity increased in the hours immediately after skin cleansing and persisted for the remainder of the study. In general, the active treatment noticed a marginal increase in diversity except at the seventh day. Evaluation of beta diversity showed no major differences across time or treatment (Figures S3–S5).

### 3.2. qPCR Staphylococcus Epidermidis and Cutibacterium Acnes

**Figure 2:**
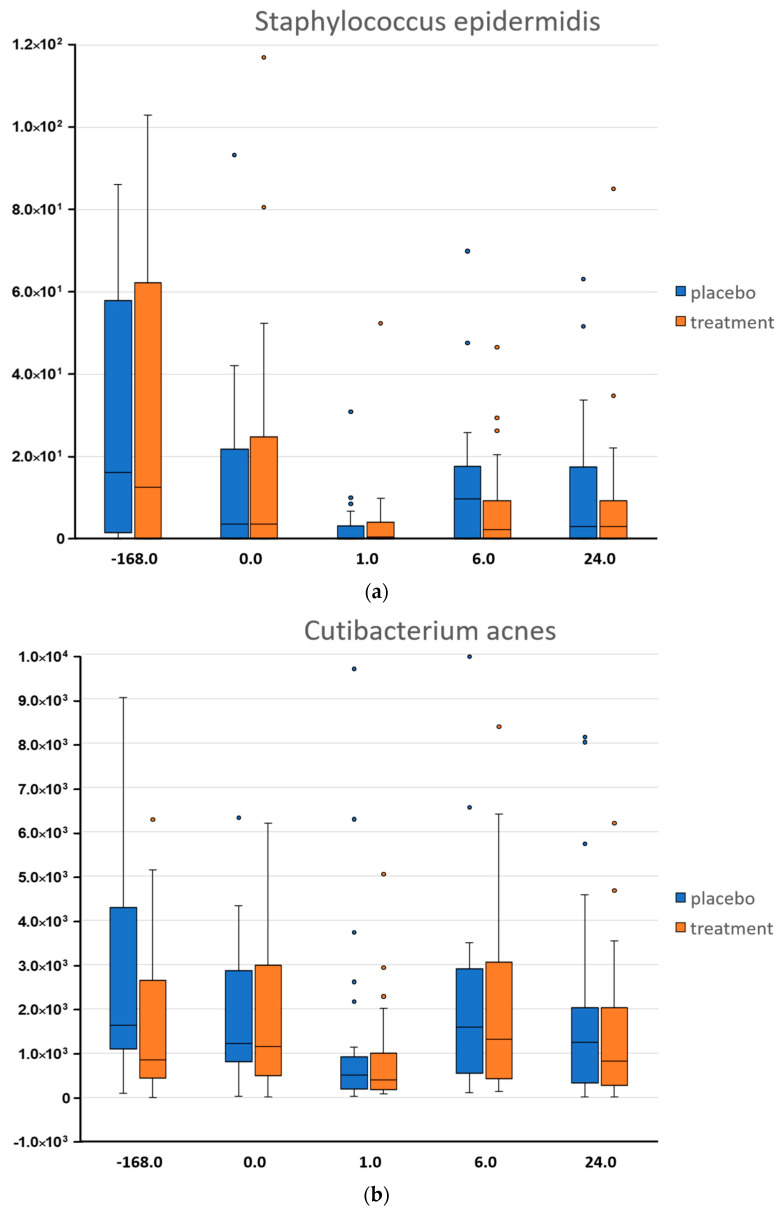
Quantification of two selected bacteria by qPCR (**a**) *Staphylococcus epidermidis* and (**b**) *Cutibacterium acnes*. Data show the distribution of each microorganism across time (h) and as a consequence of two treatments, placebo (blue) and active treatment (orange).

*Staphylococcus epidermidis* was present at its highest abundance prior to the washout phase (Figure 2). Interestingly, *S.epidermidis* and *C.acnes* abundances were normalized at T0 most likely because of the gentle cleanser used by all the study participants during the washout period. At 1 h after the first cleansing using the test body wash formulations, the two species showed a decrease which was independent of the active treatment but likely due to the cleansing procedure. At 6 h following cleansing we could observe a growth of the two bacteria, an attempt to re-establish their abundances.

### 3.3. Reference Frames

When dealing with 16S rRNA sequencing data and consequently with relative abundances comparing ratios of taxa can circumvent the bias introduced by unknown microbial loads. However, the choice of taxa for comparison between thousands of taxa might be challenging and could lead to either false-positives or false-negatives. Songbird described in Morton et al. [23] provides a way to rank microbes that are changing the most relative to each other by adopting multinomial regression to calculate differentials. The latter referring to the logarithm of the fold change in abundance of a taxa compared to others between two conditions. When relative abundance data are used, the output is a relative differential. Those differentials can be ranked to determine which taxa are changing the most between samples. This procedure is referred to as differential ranking. However, to be able to infer changes in abundance the choice of reference frames is required. In brief, “Reference frame” is built on the concept from physics where velocity is measured relative to another moving object. As the microbiome is in a dynamic equilibrium, and in absence of quantitative information on total microbial load, we can infer changes in microbial populations only relatively to reference frames given by other microbial populations. The denominator in a log-ratio determines the reference frame for inferring changes. Using the “reference frames” approach, the top 10% of features identified in the ranking were used to describe the intercept, time, and treatment for all comparisons of interest. During the washout period, the only comparison was on the intercept and time to characterize how different the microbiotas were between the two groups (intercept) and whether they diverged (time) over the washout period.

Songbird was used to understand any preliminary bias in the cohorts by evaluating the top 10% of microbiota that changed with time and examining the effect on the treatment groups. By looking at the intercept and at the relationship to time, no significant differences were observed in the additive natural log-ratio between the treatment groups (Figure S6). 

**Figure 3:**
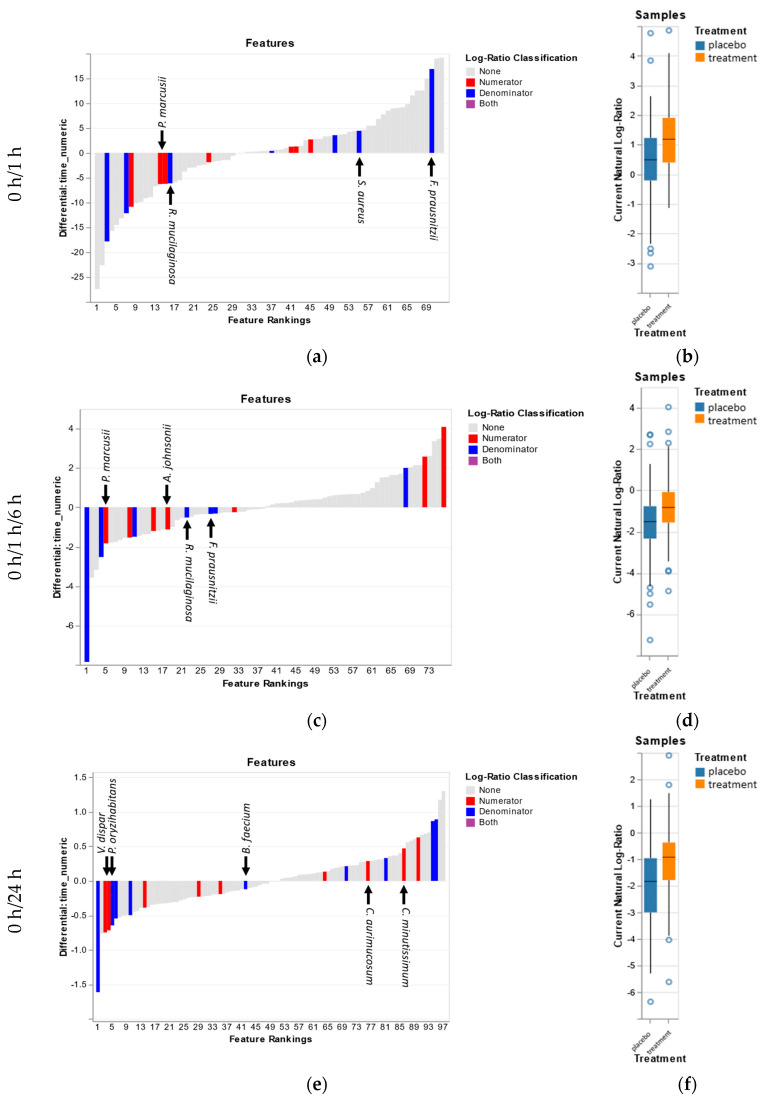

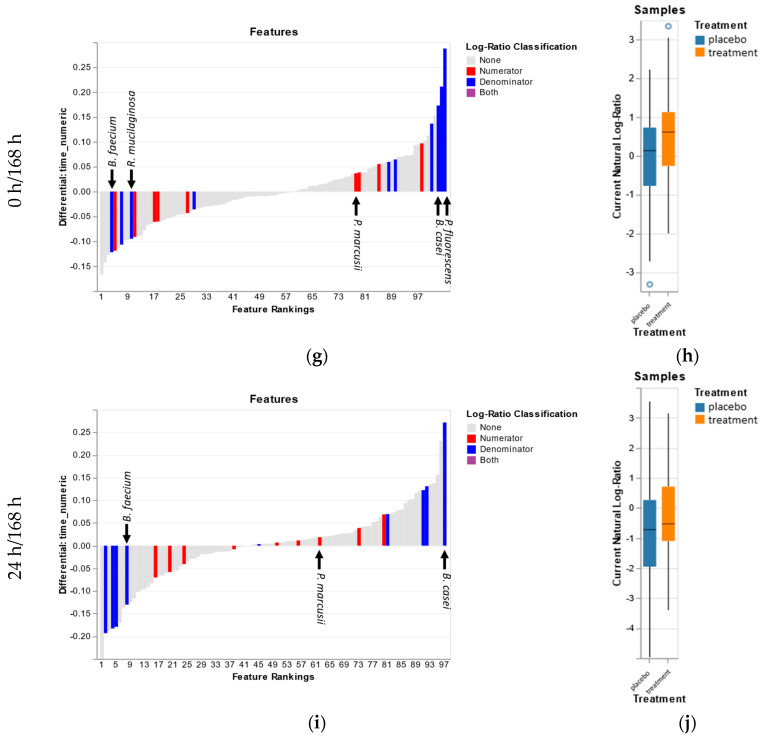
The rank plot (**a**,**c**,**e**,**g**,**i**) of the top 10% of organisms that contribute to the treatment/placebo differential plotted against their respective ranking against time. The effect of the treatment on the top 10% of contributing organisms is visualized as a boxplot (**b**,**d**,**f**,**h**,**j**).

The differential approach facilitated by songbird was applied to understand how the addition of saccharide isomerate would impact the microbiota. Examining the treatment differential relative to the time differential allows us to look at how these two parameters are associated with the same microbiota (Figure 3). Over the first hour, it can be seen that the organisms associated with the different treatments both tend to have a negative association with time (Figure 3a). This is somehow expected and it is most likely due to the cleansing procedure which is intended to remove dirt and microbiota, preferably pathogens, from the skin surface. Positive associations with time do not necessarily indicate positive growth but can be representative of microbiota that are harder to remove. Some of the bacteria negatively influenced by the active treatment over the first hour are *Staphylococcus aureus*, *Streptococcus sanguinis*, *Faecalibacterium prausnitzii*, and *Rothia mucilaginosa*. Of these, *F. prausnitzii* and *S. aureus* have a positive association with time. Several bacteria of interest that are positively associated with the active treatment are *Paracoccus marcusii* and *Acinetobacter johnsonii*. *A. johnsonii* also has a slight positive association with time. Of note is that *C. acnes*, not pictured, has a mostly neutral association with time and a slight negative association with the active treatment.

Between T0 and day 1, it is seen that the active treatment differential is starting to deviate from the time differential (Figure 3e). *Veillonella dispar* and *Pseudomonas oryzihabitans* were positively associated with the active treatment but negatively associated with time. Of note is that *Aerococcus viridans*, a Firmicutes, that decreases with time and by the active treatment at day 1, was seen decreasing over the first six hours after the washing as well. In contrast, by day 7, microbiota negatively associated with the active treatment were positively associated with time (Figure 3g). The major microbiota contributing to this contrast are *Pseuodomonas fluorescens, Brevibacterium casei,* and *Rothia mucilaginosa*. Four microbiotas were positively associated with the active treatment and time: *Actinomyces oris, Streptococcus cristatus, Actinomyces naeslundi,* and *Paracoccus marucsii*. Days 1 to 7 differentials also showed a negative association of the active treatment with *P. fluorescens* and *B. casei* (Figure 3i). We see a minor positive association of *S. cristatus* with both time and the active treatment. Here, it is seen that *Brachybacterium faecium* decreases both with time and due to the active treatment.

## 4. Discussion

The skin is an ecosystem in a delicate and dynamic balance between the skin microbiota and the host. The external environment constantly challenges this dynamic equilibrium and its resilience, intended as the ability to counteract and respond to or recover from external perturbations, is paramount for the maintenance of healthy skin [28]. Skin cleansing is part of our daily routine aiming at keeping the skin clean from dirt and pollutants as well as to prevent infections. Presumably, this activity has a direct impact on the cutaneous ecosystem and the cleansing product used has an impact, too. Extensive research describes the effects of skin cleansing on the skin biophysical properties [1,3,7] and a recent studies showed that beauty products alter molecular and bacterial diversity [29,30]. However, to the best of our knowledge no published study to date has addressed the impact of skin cleansing on the skin microbial composition.

In this study, we aimed to assess the influence of a body wash formulation (liquid soap) on the skin microbiota. We focused on the stability of the microbial composition over time after skin cleansing and we wanted to capture cleansing-associated compositional shifts and identify differentially abundant taxa associated with the active ingredient saccharide isomerate.

### 4.1. Diversity and Relative Abundance

Alpha diversity during the washout phase (the 7 days prior to the body wash formulation testing), was lower than during the next 7 days of the clinical study (Figure S2) and is likely due to the body wash composition being used during each phase of the trial. Interesting to note, is that after 1 h of the first use of the body wash, the alpha diversity increased relative to T0, suggesting a rapid colonization or, more likely, some mechanism of translocating bacteria to the skin surface. According to recent studies, each individual emits between 10^6^ to 10^7^ skin-associated microbes per hour, resulting in a significant deposition of biomass in the built environment [17,18]. Therefore, the increase in diversity we observed may be a result of an intermixing of trial subjects sharing their personal and unique “microbial cloud”, and/or the mixing of people within built environment. The shift in diversity likely comes from a decrease in the genus Staphylococcus (Figure 1). After the washout phase, no significant changes in alpha diversity were observed due to treatment.

The washout phase is considered a period of time in which study participants discontinue their previous treatments/topical cosmetic applications before the study actually begins. In this study, during a seven-day period the volunteers were allowed to use only a gentle cleanser which was defined in the study protocol and was the same for all the study participants. Interestingly, we found that the washout phase, had already an impact on the skin microbiome. Particularly, at the genus level we saw a reduction in *Staphylococcus* which may be due to the gentle cleanser usage itself as the largest presence of *Staphylococcus* was in the constituents prior to the start of the trial. The qPCR analysis was performed to quantify the amount of DNA of two specific bacteria, which are well-known skin commensals: *Staphylococcus epidermidis* and *Cutibacterium acnes* (Figure 3). The quantitative data perfectly matched with the decrease in *Staphylococcus* relative abundance and additionally, revealed a reduction of *Staphylococcus epidermidis* at T0 after the washout phase. At T0, while *Staphylococcus* was still the most abundant genus, there was a large increase in the relative abundance of the genus *Micrococcus*. The two major species of *Micrococcus* observed were *M. terreus* and *M. luteus*, but the observation of *M. terreus* could easily be a misclassification of such a short 16S read. *M. luteus* has been observed as part of the common skin microbiota and can easily be found among soil, air, and other environments [31]. The 1 h time point showed a minor decrease in the Micrococcus genus, but no significant changes were observed among the treatments, suggesting that despite Micrococcus’s ability for rapid growth, it is likely nutrient limited.

At 1 h after the skin cleansing we expected to see a decrease in the microbial load due to the rinse-off procedure, and as expected, a decrease in abundance of *S.epidermidis* and *C.acnes* was observed. At 6 h past the first test products application, an increase in *C. acnes* was observed compared to 1 h despite *C. acnes* being considered a slow-growing bacteria with a doubling time of 5.1 h [32]. However, qPCR data supports the idea that in just 6 h post cleansing, there is rapid growth of *C. acnes*. Alternatively, a decrease in observed organisms over the first six hours may more realistically represent what bacterial populations were removed and/or detached from the skin surface due to the action of the body wash as we expect a decrease in total population of which the data is not captured in NGS data. By day one into the study, the microbiota appears to be restructuring as observed by an increase in the abundance of *Acinetobacter*. The major observed species of *Acinetobacter*, *Acinetobacter lwoffii* and *Acinetobacter johnsonii*, were not surprising and represent common skin microbiota that may protect against allergic sensitization and inflammation [33,34].

After seven days of using the body wash, while no significant (*p* ≤ 0.05) differences were observed at the genus level between the treatment and control groups, 80 species had significant differences prior to family-wise error correction. Within these 80 species, *Stapyhlococcus succinus*, *Corynebacterium glutamicum*, and *Rothia mucilaginosa* were of interest either because of the relevance of their parent genus or because they were found via the reference frames method. *S. succinus* was increased in the active treatment (*p* = 0.009) and may be an important immune modulator [35]. *C. glutamicum* was decreased in the active treatment and is considered to be part of a group of “coryneform” bacteria that are increasingly implicated in significant infections [36]. The reduction of this and other detected “coryneform” bacteria using the active treatment may play a role in day-to-day management of opportunistic infections from this group. *R. mucilaginosa* was increased (*p* = 0.022) in the active treatment and has been implicated in wide range of serious infections [37]. This finding of *R. mucilaginosa* is in direct contrast to what was found in the use of “reference frames” for evaluating association of microbiota to the active treatment. As the current methods to study the skin microbiome based on relative abundance of microbes are prone to high false discovery rates [23], the new methodology applied herein constitutes a novel and successful analytical approach to assess microbiome datasets for the personal care industry.

### 4.2. Reference Frames

Using “reference frames”, while still a new and novel approach to evaluating microbial associations, represents an advancement in techniques that shows promise in handling compositional data and may improve the conclusions we draw. While the “reference frame” approach does not designate p-value associations for differential abundance, it shows “greater ability to identify correct taxa” [38,39]. Therefore, it was interesting to apply Songbird to a low-biomass dataset such as the skin microbiota as the test case in the original article had explored a similar low-biomass environment [23]. Using Songbird firstly provided us a way of deconvoluting the effect of both time and treatment by building a multinomial regression model that can convey not just if there was a difference, but build a full model to describe the changes. With sampling points that convey distinct segments throughout the whole trial (washout, immediately after washing, day after, and stable use), Songbird was applied to the different segments to deconvolute the effects of the active treatment during these intervals (Figure 3). Some of our results were validated using qPCR on specific taxa (*S. epidermidis*, *C. acnes*) and aided in our confidence of Songbird’s ability to detect appropriate associations.

During the first 6 h after first use of the active treatment, Songbird picked up interesting trends that were also validated by qPCR, such as an increase in *C. acnes*. The sharp increase in *F. prausnitzii*, a common gastrointestinal organism, likely due to hygiene issues during bathroom use and/or the microbial cloud was interesting [17]. Whereas, in opposition, a decrease in *A. lwoffii* is likely due to the washing of the skin and subsequent removal of the microbiota. The model also provides evidence that while the placebo body wash is adept at the removal of *R. mucilaginosa*, adding the active body wash formulation further decreased the presence of this “coryneform”. Likewise, the active formulation is better at the removal of *S. aureus* and *F. prausnitzii* than the placebo but the natural repopulation of these bacteria on the skin surface occurs due to another factor. By 6 h after washing, the growth of *C. acnes* was observed despite a noted “slow-growing” expectation [32]. This growth does fit within the 5.1 h doubling time but indicates something more interesting; *C. acnes* is both in an active growth state and not completely removed during the use of the wash formulations, likely due to the fact that it resides also within skin pores and sebaceous glands, locations where the action of a skin cleanser is limited.

By 24 h, adaptation to the new body formulation appears to have taken place. When comparing the top 10% of microbiota contributing to the time differential of both the T0 versus 24 h and T0 versus 168 h, 50% of the organisms negatively associated with time are the same. At 24 h, the active formulation is negatively associated with bacteria such as *Brachybacterium faecium* and *Brevibacterium casei*. Both of these bacteria are recognized as opportunistic and able to cause diseases and, therefore, improved removal of these microbiota can potentially mitigate infections in certain subjects [36]. By 168 h, a similar trend in the removal of these “coryneforms” by the active treatment is observed and represent a previously uncharacterized benefit of the active wash formulation. Of potentially beneficial organisms, *Paracoccus marcusii* was positively associated with the active formulation. As at 24 h, several microbiotas had been removed, examining the differential ranking between 24 and 168 h reflects any ongoing enrichment of microbiota due to the subtleties of the active wash. Again, the active wash formulation removes the “coryneforms” and enriches for *P. marcusii*.

Despite the interpersonal variability of the skin microbiota, *Paracoccus marcusii* has been identified as a taxon positively associated with the active treatment at all the timepoints evaluated. Interestingly, this species is known to be a natural producer of astaxanthin, a xanthophyll carotenoid, regarded as a potent antioxidant which has potential health-promoting effects in the prevention or treatment of various diseases, ranging from cancers to diabetes. It has been shown that it has as well skin protective effects such as a pronounced photoprotective effect by counteracting ultraviolet A-induced oxidative damage [40,41]. Other studies suggested that topical or oral administration of astaxanthin might be helpful to prevent or to significantly reduce the negative effects of UV-A exposure such as skin sagging or wrinkling [42,43]. However, astaxanthin like all carotenoids is a natural pigment with a strong color which makes it difficult to be incorporated into a cosmetic formulation without impacting the color of the final product. Furthermore carotenoids are also known to be very unstable compounds being sensitive to light, temperature and extreme pH in the presence of oxygen [44]. In our study, we surprisingly have found, via the “reference frames” approach, that the body wash formulation containing saccharide isomerate led to a selective and consistent increase in the relative differential abundance of *P. marcusii* which is a highly interesting outcome seeing the many skin benefits attributed to its metabolic product astaxanthin. Interestingly, besides being a known carotenoid producer, *P. marcusii* has been reported to be a promising hydrocarbons degrader, especially a degrader of polyaromatic hydrocarbons (PAHs) and a producer of biosurfactants [45,46]. PAHs are known to be adsorbed onto the skin as a result of environmental pollution and are potentially carcinogenic. Therefore, a certainly interesting microorganism with great abilities to degrade, detoxify, and render contaminants harmless. For all these reasons, *P. marcusii*, an up to know overlooked skin commensal, might represent a key organism for health looking skin and we believe it deserves more attention in the near future.

When studying the skin microbiome, several study limitations may arise mainly linked to the complexity of this fast-developing research topic. The skin microbiome’s overall composition is known to vary greatly depending on the body site; however, although our study was limited to the volar forearm we have evidence (data not shown) of the presence of the taxa mentioned above also on face of individuals. The volar forearm was chosen as sampling site for several reasons: (1) it is a body site with high microbial diversity; (2) it is a dry skin type therefore useful to see if the product tested brings any moisturizing benefits; (3) being the product a body wash it is meant to be applied on body and the volar forearm is one of the standard testing sites for cosmetic efficacy studies. Age is also known to have an impact on the skin microbiota composition and diversity which go along with skin biophysical changes. We deliberately decided to focus our attention on healthy females in their 20s and 40s and we excluded the population in which hormonal changes due to puberty or post-menopause phases would increase variability in an already complex topic like the skin microbiome.

## 5. Conclusions

This study revealed cleansing-associated microbial changes and allowed to assess the beneficial effects of saccharide isomerate on skin. Despite the short as well as long-term perturbation because of skin cleansing, the skin microbiota proved to be resilient and able to re-establish itself and to adapt its composition. The “reference frames” method employed to analyze the NGS data allowed to deconvolve the effect of both time and treatment to infer changes associated with the active treatment that included saccharide isomerate. The active body wash provided potential interesting benefits by reducing so called “coryneforms” such as *B.casei* and *R.mucilaginosa*, bacteria which are increasingly implicated in skin infections. On the other hand, the beneficial bacterium, *P.marcusii* was shown to be enriched when the active formulation was used. In the future, metagenomic studies will certainly be needed to help dive deeper into the functionality of these microbial taxa and establish a clear relationship with a certain, specific skin phenotype.

## Figures and Tables

**Table 1:** Formulations used in the clinical study.

	Base Formulation (Placebo)	Active Formulation
INCI Name	%	%
Aqua	57.50	57.00
Disodium EDTA, aqua	0.05	0.05
Lauryl glucoside	4.00	4.00
PEG-32	5.00	5.00
Lauric acid	3.30	3.30
Stearic acid	12.00	12.00
Myristic acid	11.50	11.50
Glyceryl stearate, PEG-100 stearate	2.00	2.00
Potassium hydroxide	4.65	4.65
Saccharide isomerate, aqua, citric acid, sodium citrate	0.00	0.50
Total	100	100

## Supplementary Materials

The following are available online at https://www.mdpi.com/2076-2607/8/11/1634/s1, Figure S1. Study timeline and microbiome sampling timepoints. Figure S2. Alpha diversity using Shannon’s entropy. Figure S3. Bray–Curtis measure of beta-diversity between time 0 and 6 h after the first use of the active treatment. Figure S4. Bray–Curtis measure of beta-diversity between time 0 and 24 h after the first use of the active treatment. Figure S5. Bray–Curtis measure of beta-diversity between time 0 and 168 h after the first use of the active treatment. Figure S6. The top 10% of OTUs in the differential rankings produced by Songbird for the placebo and treatment groups during the washout phase.
