# Supplementary material for: Microbial Reference Frames Reveal Distinct Shifts in the Skin Microbiota after Cleansing
Source: Microorganisms. 2020 Oct 23;8(11):1634. doi: 10.3390/microorganisms8111634 (PMC7690701; doi:10.3390/microorganisms8111634)
Supplement: Supplementary file 1 [file microorganisms-08-01634-s001.pdf]

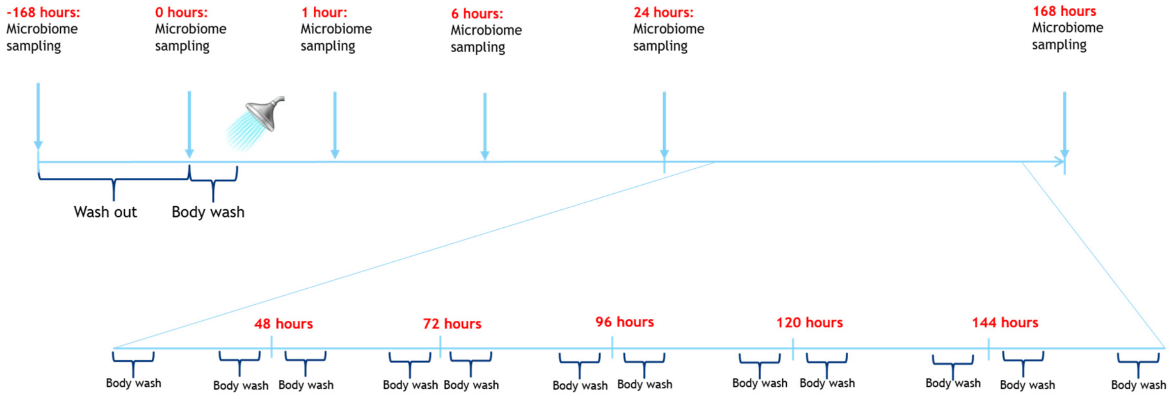

Figure S1. Study timeline and microbiome sampling timepoints.

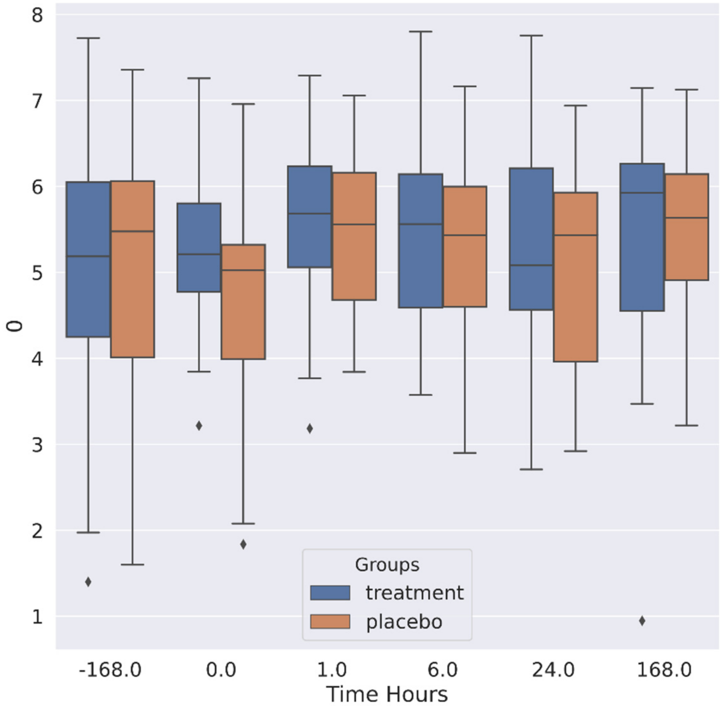

Figure S2. Alpha diversity using Shannon's entropy. At time -168 h represents subjects prior to washout and at time 0 h represents subjects having used the same formulation for washout and therefore grouping is symbolic for these timepoints to describe how the two groups change independent of the active treatment.

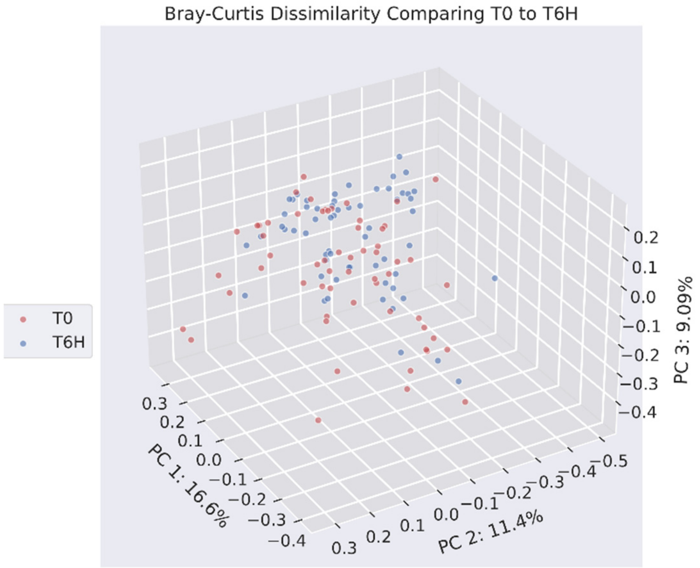

Figure S3. Bray-Curtis measure of beta-diversity between time 0 and 6 h after the first use of the active treatment. Treatment compared to control was not evaluated as no large differences between the groups were expected.

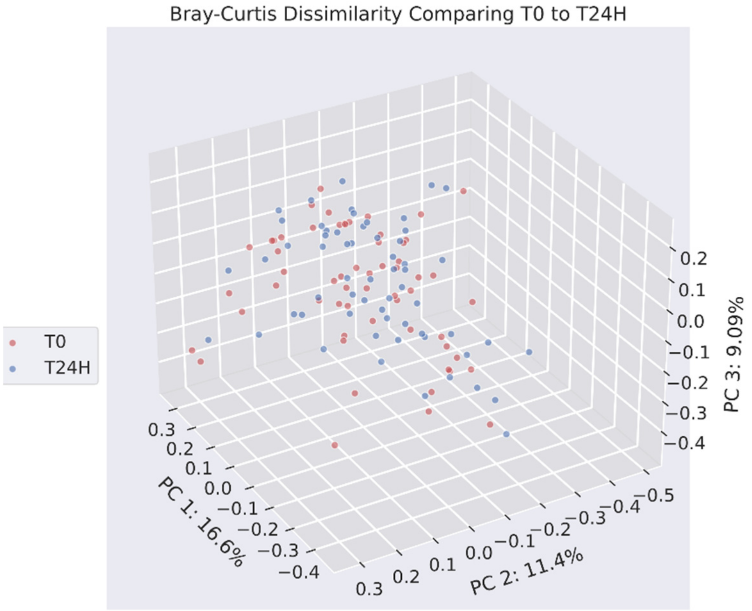

Figure S4. Bray-Curtis measure of beta-diversity between time 0 and 24 h after the first use of the active treatment. Treatment compared to control was not evaluated as no large differences between the groups were expected.

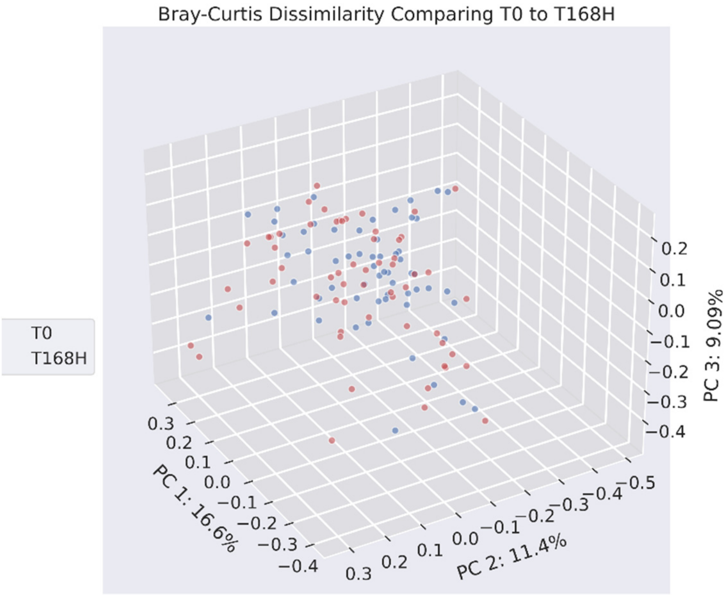

Figure S5. Bray-Curtis measure of beta-diversity between time 0 and 168 h after the first use of the active treatment. Treatment compared to control was not evaluated as no large differences between the groups were expected.

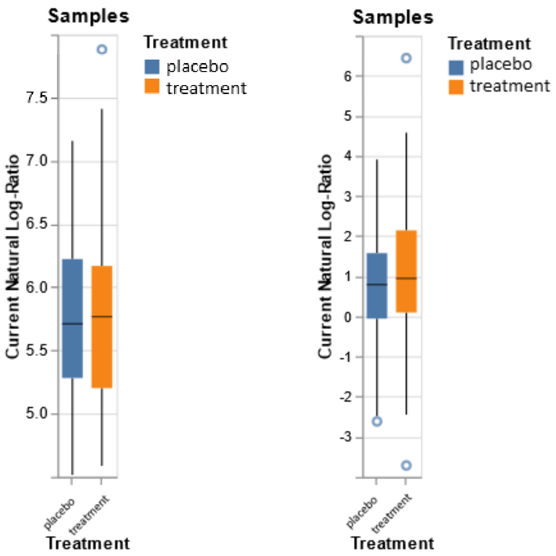

(**a**) (**b**)

Figure S6. The top 10% of OTUs in the differential rankings produced by Songbird for the placebo and treatment groups during the washout phase. (**a**) is the intercept representing the starting point;(**b**) is the time ranking and represents any divergence of the groups during washout.
